# Physiological carry-over effects of variable precipitation are mediated by reproductive status in a long-lived ungulate

**DOI:** 10.1093/conphys/coae045

**Published:** 2024-07-05

**Authors:** Joseph A Hediger, Bryan D Spencer, Michaela F Rice, Miranda L Hopper, Randy W DeYoung, J Alfonso Ortega-Santos, Timothy E Fulbright, David G Hewitt, Aaron M Foley, Landon R Schofield, Tyler A Campbell, Michael J Sheriff, Michael J Cherry

**Affiliations:** Caesar Kleberg Wildlife Research Institute, Texas A&M University –Kingsville, 700 University Boulevard, Kingsville, TX 78363, USA; Caesar Kleberg Wildlife Research Institute, Texas A&M University –Kingsville, 700 University Boulevard, Kingsville, TX 78363, USA; Minnesota Department of Natural Resources, 500 Lafayette Road North, Saint Paul, MN 55155, USA; Caesar Kleberg Wildlife Research Institute, Texas A&M University –Kingsville, 700 University Boulevard, Kingsville, TX 78363, USA; Caesar Kleberg Wildlife Research Institute, Texas A&M University –Kingsville, 700 University Boulevard, Kingsville, TX 78363, USA; Caesar Kleberg Wildlife Research Institute, Texas A&M University –Kingsville, 700 University Boulevard, Kingsville, TX 78363, USA; Caesar Kleberg Wildlife Research Institute, Texas A&M University –Kingsville, 700 University Boulevard, Kingsville, TX 78363, USA; Caesar Kleberg Wildlife Research Institute, Texas A&M University –Kingsville, 700 University Boulevard, Kingsville, TX 78363, USA; Caesar Kleberg Wildlife Research Institute, Texas A&M University –Kingsville, 700 University Boulevard, Kingsville, TX 78363, USA; East Foundation, 200 Concord Plaza Drive, Suite 410, San Antonio, TX 78216, USA; East Foundation, 200 Concord Plaza Drive, Suite 410, San Antonio, TX 78216, USA; Biology Department, University of Massachusetts-Dartmouth, 285 Old Westport Road, Dartmouth, MA 02747, USA; Caesar Kleberg Wildlife Research Institute, Texas A&M University –Kingsville, 700 University Boulevard, Kingsville, TX 78363, USA

**Keywords:** Glucocorticoids, lactation, legacy effects, rainfall, reproductive trade-off, white-tailed deer

## Abstract

In the age of global climate change, extreme climatic events are expected to increase in frequency and severity. Animals will be forced to cope with these novel stressors in their environment. Glucocorticoids (i.e. ‘stress’ hormones) facilitate an animal’s ability to cope with their environment. To date, most studies involving glucocorticoids focus on the immediate physiological effects of an environmental stressor on an individual, few studies have investigated the long-term physiological impacts of such stressors. Here, we tested the hypothesis that previous exposure to an environmental stressor will impart lasting consequences to an individual’s glucocorticoid levels. In semi-arid environments, variable rainfall drives forage availability for herbivores. Reduced seasonal precipitation can present an extreme environmental stressor potentially imparting long-term impacts on an individual’s glucocorticoid levels. We examined the effects of rainfall and environmental characteristics (i.e. soil and vegetation attributes) during fawn-rearing (i.e. summer) on subsequent glucocorticoid levels of female white-tailed deer (*Odocoileus virginianus*) in autumn. We captured 124 adult (≥2.5-year-old) female deer via aerial net-gunning during autumn of 2015, 2016 and 2021 across four populations spanning a gradient of environmental characteristics and rainfall in the semi-arid environment of South Texas, USA. We found for every 1 cm decrease in summer rainfall, faecal glucocorticoid levels in autumn increased 6.9%, but only in lactating females. Glucocorticoid levels in non-lactating, female deer were relatively insensitive to environmental conditions. Our study demonstrates the long-lasting effects of environmental stressors on an individual’s glucocorticoid levels. A better understanding of the long-term effects stressors impart on an individual’s glucocorticoid levels will help to evaluate the totality of the cost of a stressor to an individual’s welfare and predict the consequences of future climate scenarios.

## Teaser

Past experiences may impart lasting effects on an individual despite temporal separation from the inciting event. Through an unmanipulated field study across 4 populations of free-living, white-tailed deer, we investigated the effect of variable summer rainfall on fecal glucocorticoid metabolites in adult females in autumn. We found evidence reduced summer rainfall imparted long-term effects on female glucocorticoid levels, but the effect of past environmental conditions depended on reproductive status.

## Introduction

Individuals constantly interact with and respond to threats in their environment (Sheriff *et al.*, 2010; Borowik *et al.*, 2020; Abernathy *et al.*, 2022). While some threats may be escapable via habitat selection and movement [e.g. predation (Smith *et al.*, 2019; Dickie *et al.*, 2020); extreme storms (Abernathy *et al.*, 2019); local drought (Abraham *et al.*, 2019)], other threats occur at broader geographic extents and may be inescapable [e.g. shifting competitive balances (Poloczanska *et al.*, 2008); thermal dysregulation (Lenarz *et al.*, 2009); reduced food availability (Wasser *et al.*, 2017)]. With global climate change, such inescapable threats, including broad-scale drought, are predicted to increase in severity and frequency (Smith, 2011; Spinoni *et al.,* 2018; IPCC, 2021). Therefore, animals will increasingly be forced to cope with such changes in their environment (Monteith *et al.*, 2015; Sydeman *et al.*, 2015; Ruprecht *et al.*, 2016). Fitness-related consequences to such events can occur immediately in response to the stressor but may persist long after the stressor has occurred, inducing a carry-over effect (Harrison *et al.*, 2011; Legagneux *et al.*, 2012; Blomberg *et al.*, 2014). Here, we define carry-over effects as a past experience imparting an effect on an individual despite temporal separation between the inciting event and the impact (Harrison *et al.*, 2011). For example, eastern fence lizards (*Sceloporus undulatus*) treated with exogenous stress hormones had reduced fitness (i.e. reproductive success and survival); however, the effect on fitness was exacerbated when females also experienced higher ambient temperatures the previous winter due to increased energy expenditure during hibernation (MacLeod *et al.*, 2018). While laboratory studies have begun to identify physiological mechanisms associated with carry-over effects (Banerjee *et al.,* 2011; Taborsky *et al.*, 2013; Ledón-Rettig *et al.*, 2023), such studies are rare in free-living mammals (Davies *et al.*, 2013; Davy *et al.*, 2017).

One of the most conserved physiological responses to environmental stressors in vertebrates is the activation of the hypothalamic–pituitary–adrenal (HPA) axis and subsequent release of glucocorticoids (GC) (Sapolsky *et al.*, 2000; Sheriff *et al.*, 2009; Bonier *et al.*, 2009a). When an individual perceives a stressor, the hippocampus triggers the activation of the HPA axis thus increasing GC secretion from the adrenal glands (Sapolsky *et al.*, 2000; Herman *et al.*, 2005). Physiologically, GC secretion increases plasma glucose levels by decreasing the use of glucose for non-essential processes and towards energetically demanding processes (MacDougall-Shackleton *et al.*, 2019). While GCs are often termed ‘stress’ hormones, GCs better represent a mediator of energetic balance within an individual (Sapolsky *et al.*, 2000; Remage-Healey and Romero, 2001; Landys *et al.*, 2006). GCs trigger a number of physiological responses leading to a cascade of downstream effects shifting energy expenditure in the body to meet the energetic demands the stressor imposes on the individual (Wingfield *et al.*, 1998; Charmandari *et al.*, 2005). These responses to stressors are specific to the individual and may mediate energetic trade-offs (e.g. reproduction, survival, body condition) or drive changes in behaviour (e.g. increase foraging or refuge use) to optimize fitness (Bonier *et al.*, 2009b; Boonstra, 2013). For example, many studies have found GCs are associated with reduced reproductive output in favour of increased survival (Wingfield *et al.*, 1998; Sapolsky *et al.*, 2000; Lancaster *et al.*, 2008; Sheriff *et al.*, 2009; Almasi *et al.*, 2013; Patterson *et al.*, 2014; MacLeod *et al.*, 2018), while other studies have demonstrated increased GCs may help individuals maintain reproductive output when experiencing a stressor, but at the cost of body condition (e.g. Sheriff *et al.*, 2017). GCs are commonly quantified through faecal GC metabolites (FGM) because of the rapid and extensive metabolism of plasma GCs before excretion (Wasser *et al.*, 2000). While we are gaining a better understanding of how environmental stressors may impact GCs and the potential outcomes of such responses, little is known about the long-term impacts of past stressors on current GC levels in a long-lived, free-living mammal.

The role GCs play in mediating energetic trade-offs in response to environmental stressors suggests GCs may be integral in our understanding of the long-term impacts of past stressors on individuals. GCs are elevated in response to a stressor (Sapolsky *et al.*, 2000; Charmandari *et al.*, 2005). If an individual is currently coping with or recovering from a previous stressor, GCs may mediate this effect. For example, in koalas (*Phascolarctos cinereus*), rainfall two months prior to sample collection was predictive of current GC levels, an effect attributed to vegetation growth and leaf moisture (Davies *et al.*, 2013). Through better understanding of the long-term impacts of stressors on individuals, we can evaluate the totality of effects a stressor has on an individual.

Drought is one of the most important environmental stressors that is increasing in frequency and intensity with changing climate (Gesquiere *et al.*, 2008; Carrão *et al.*, 2018; Naumann *et al.*, 2018; Anderwald *et al.*, 2021), especially in arid and semi-arid regions where climate change is expected to increase desertification (Sherwood and Fu, 2014). Desertification imparts severe consequences to wildlife in these regions given the importance of rainfall for plants and forage availability (Peterson *et al.*, 1992; Lidon, 2012; Folks *et al.*, 2014; Cain *et al.*, 2017; DeYoung *et al.*, 2019). For example, forbs, a critical nutritional resource for herbivores, are mostly absent during periods of drought in many semi-arid grasslands (DeYoung *et al.*, 2019). Most studies of the effects of drought on GCs evaluated the short-term immediate effects (Strier *et al.*, 1999; Foley *et al.*, 2001; King and Bradshaw, 2010), while comparatively few studies have explored how drought may impart long-term consequences to an individual’s GC levels recovering from past drought (Davies *et al.*, 2013). Understanding the physiological mechanism allowing free-living animals to cope with a previous environmental stressor (i.e. drought) is essential, especially when arid climates are predicted to replace more temperate climates (Alessandri *et al.*, 2014).

The group of animals most likely to be impacted by drought are those who are most energetically vulnerable (e.g. juveniles, pregnant females, lactating females). For example, rainfall events occurring in mid- to late-pregnancy had the strongest positive effect on subsequent juvenile survival and recruitment in mule deer (*Odocoileus hemionus*) populations (Heffelfinger *et al.*, 2018). An effect attributed to rainfall and subsequent forage availability during lactation, which accounts for upwards of 80% of total energetic costs during reproduction due to increased water and dietary requirements (Jönsson, 1997; Pekins *et al.*, 1998; Wheatley *et al.*, 2008). Subsequently, the most energetically vulnerable individuals may remain in an energy deficit long after the drought has passed. To mediate an energetic balance, GCs may remain elevated long past resolution of the drought.

**Figure 1:**
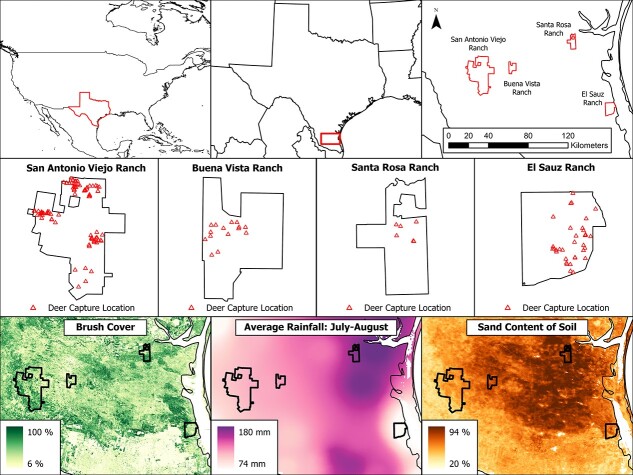
Maps displaying our study areas in South Texas, USA encompassing four East Foundation ranches: Buena Vista Ranch, El Sauz Ranch, Santa Rosa Ranch and San Antonio Viejo Ranch. Top row: Sequentially smaller showing the geographic location of the study ranches; Middle row: Distribution of captured deer GPS locations by ranch; Bottom left: Spatial representation of percentage of brush cover; Bottom centre: Spatial representation of total summer rainfall (mm) averaged across 2015, 2016 and 2021; Bottom right: Spatial representation of percentage of sand content of the soil. The areas of high sand content demonstrate the ecoregion of the Coastal Sand Plain.

Here, we tested two competing hypotheses. The first being previous exposure to an environmental stressor would have lasting consequences to an individual’s GC levels. The second being current conditions are more predictive for an individual’s GC levels regardless of previous exposure to environmental stressors. To test these hypotheses, we examined how variable summer and autumn rainfall influenced the FGM concentrations of free-living, female white-tailed deer (*Odocoileus virginianus*) in autumn. To properly estimate the rainfall effect, we included environmental characteristics (i.e. percent sand in the surface soils, percent brush cover) to account for spatial variation in habitat quality. Our study occurred in a semi-arid ecosystem in South Texas, USA on four ranches that span a gradient in annual rainfall and environmental characteristics (Fig. 1). In South Texas, summer is characterized by highly variable rainfall and temporally overlaps the life stages of white-tailed deer with the highest energetic demands (i.e. late gestation, early lactation). Therefore, individuals are likely to encounter highly variable precipitation during this time, potentially inducing carry-over effects.

We predicted that (i) reproductively mature female deer exposed to reduced summer rainfall would have higher FGM concentrations in autumn, (ii) this effect would be greatest in those females carrying the largest energetic costs (i.e. lactating females) and (iii) this physiological carry-over effect would influence FGM concentrations more than current conditions due to the high demands of maternal investment during this critical life-history period. To discern between a long-term effect of reduced summer rainfall and an immediate effect, we examined the influence of current environmental conditions (i.e. percent sand in the surface soils, percent brush cover, rainfall 1-month and 2-months prior to capture—all strong predictors for habitat quality and vegetation growth (Davies *et al.*, 2013; Foley *et al.*, 2018; Rankins *et al.*, 2023b) within the study area on female FGM concentrations. Our study fills a critical knowledge gap examining how a past environmental stressor may influence FGM concentrations in a long-lived, iteroparous ungulate in an unmanipulated field study.

## Materials and Methods

### Study area

This research was conducted in the South Texas Plains and Coastal Sand Plain ecoregions (Supplementary Material Fig. S1 and Fig. S2) of Texas, USA (Bailey, 1995) during the years 2015, 2016 and 2021. We conducted research on four East Foundation ranches: Buena Vista Ranch (6113 ha), El Sauz Ranch (10 984 ha), Santa Rosa Ranch (7544 ha) and San Antonio Viejo Ranch (60 752 ha). East Foundation lands are utilized to promote the advancement of land stewardship through ranching, science and education. As such, these lands are working cattle ranches. Native wildlife are not hunted, and the predator population is unexploited. Vegetation characteristics and rainfall patterns varied among and within sites (Fulbright *et al.*, 2021; Rankins *et al.*, 2023b, 2023a, Fig. 1). The Coastal Sand Plain consists mostly of sandier, less productive soils (i.e. decreased soil water-holding capacities) when compared to the more productive soils of the South Texas Plains (Lohse, 1952; Fulbright *et al.*, 1990, Fig. 1). Annual rainfall across the Coastal Sand Plain averages 64 cm (range 54–76 cm), while the South Texas Plains average 63 cm (range 48–91 cm) (Oregon State University, 2011, 30-year average, Supplementary Material Fig. S1).

### White-tailed deer capture and sample collection

We captured 124 reproductively mature female white-tailed deer via aerial net-gunning (Webb *et al.*, 2008) during October and November of 2015, 2016 and 2021 (Table 1). White-tailed deer in South Texas are an ideal species to test the influence of past environmental stressors on current FGM concentrations due to their life histories and reproductive strategies (Mautz, 1978; Pekins *et al.*, 1998). In our system, white-tailed deer develop fat reserves prior to the breeding season and then utilize currently available forage during late gestation and lactation to maintain their energetic requirements. In years with reduced rainfall and poor environmental conditions, female deer may finance late gestation and lactation solely on body reserves (Mautz, 1978). Due to the stochasticity in weather patterns in our system, environmental conditions experienced at ovulation (e.g. November, December), may be drastically different at parturition (e.g. June, July) (Illige, 1951; Kie and White, 1985). Therefore, to maximize the range of environmental conditions (i.e. rainfall, environmental characteristics), we distributed our sampling effort across the ranches and years (Fig. 1). Upon capture, we recorded a global positioning system (GPS) location (eTrex 10, eTrex 22x, Garmin, Olathe, KS, USA), secured the deer with hobbles, applied a blindfold and immediately transported them to a central processing area. We determined reproductive status from evidence of lactation via teat palpation and milk expression, estimated age using tooth wear and replacement (Severinghaus, 1949; Foley *et al.*, 2021), determined body condition score through visualization and palpation (Riney, 1960) and collected a faecal sample for FGM concentration analysis (Sheriff *et al.*, 2011; Dantzer *et al.*, 2014). To uniquely identify individuals, we placed numbered aluminum ear tags (style 1005–49; National Band and Tag Company, Newport, KY, USA) and released captured deer on site (Belser *et al.*, 2017). All animal handling followed protocols approved by the Texas A&M University-Kingsville Institutional Animal Care and Use Committee (protocol numbers: 2014-09-29 and 2020-10-19).

**Table 1:** Reproductive status of captured mature (≥2.5-year-old) female white-tailed deer by year and East Foundation ranch. N = total number of deer, SAV = San Antonio Viejo Ranch

Ranch	Year	*N*	Lactating	Not lactating
Santa Rosa	2015	4	1	3
Buena Vista	2015	7	4	3
Buena Vista	2016	6	6	0
El Sauz	2015	14	6	8
El Sauz	2016	13	1	12
SAV	2015	23	12	11
SAV	2016	35	10	25
SAV	2021	22	11	11
Total		124	51	73

### Environmental parameters

We obtained rainfall totals for the months of July and August of each capture year (i.e. 2015, 2016, 2021) using the raster layer produced by the PRISM Climatic Group (Oregon State University, 2011, spatial resolution: 4 km, Fig. 1, Supplementary Material Table S1). We selected July and August as these months coincide with early lactation, the most energetically costly stage of reproduction (Gittleman and Thompson, 1988; Stearns, 1992; Therrien *et al.*, 2008; Wheatley *et al.*, 2008). We also included rainfall 1- and 2-months prior to capture as these months predict current vegetation growth and habitat quality (Davies *et al.*, 2013; DeYoung *et al.*, 2019). We sourced raster layers for percent sand (spatial resolution: 250 m, Fig. 1, Supplementary Material Table S2) in the surface soil horizon (0–5 cm) from the International Soil Reference and Information Centre (Poggio *et al.*, 2021) and annual estimates of percent brush cover (spatial resolution: 30 m, Fig. 1, Supplementary Material Table S3) from the Rangeland Analysis Platform (Allred *et al.*, 2021). For each of these covariates, we averaged the conditions an animal would have experienced for a given capture year within a 150-ha buffer around each deer capture location, providing a single value for each environmental metric for each individual deer. We used 150-ha buffer as this size is a reasonable proxy of space use of deer in the system and represents an average home range size based upon a concurrent study of deer movement using GPS collared adult female deer (Spencer *et al.*, 2024). We excluded inaccessible areas (i.e. coastal bay, perimeter fence) from an individual’s buffer prior to calculating the mean values for environmental conditions within the buffers.

### Faecal collection and faecal GC metabolite analysis

To estimate GCs in white-tailed deer, we measured FGM concentrations, which represent an integrated average of plasma GC levels an individual experienced over a species-specific time duration (Sheriff *et al.*, 2011; Dantzer *et al.*, 2014). For white-tailed deer, FGMs represent an average of plasma GC levels 12–24 hours prior to collection depending upon gut passage time of the individual (Millspaugh *et al.*, 2002). Due to the integrated average of plasma GC levels with FGMs, we can determine if an individual is still coping with or recovering from the stress of reduced summer rainfall without complicating factors such as capture stress.

We collected faecal samples directly from the rectum, placed samples into individual whirl-paks and immediately placed samples into a cooler of wet ice. Within 12 hours of collection, we placed samples into a − 14*°*C freezer. Within 48 hours of collection, we placed all samples into a − 20*°*C freezer until further analysis. Detailed methods for the sample analysis can be found in Millspaugh *et al.* (2002), who validated this technique specifically for use with white-tailed deer. Briefly, we freeze-dried samples for 24 hours using a lyophilizer before we ground the samples into a homogenous powder. We then added 15 mL of 70% ethanol to a 0.1 ± 0.01 g subsample and vortexed for 30 minutes. We then centrifuged samples for 20 minutes at 2500 rpm and the resultant supernatant was stored at −20*°*C until assayed. We analysed samples using an I^125^ corticosterone radioimmunoassay kit (Catalogue No. 207120, MP Biomedicals, Costa Mesa, CA) to quantify FGM concentrations. We analysed samples according to the manufacturer’s protocol with the exception of halving the volume of all reagents (Wasser *et al.*, 2000; Millspaugh *et al.*, 2002).

### Statistical analyses

We conducted all data preparation and analysis using Program R version 4.2.2 (R Core Team, 2018). Using the ‘lme4’ package (Bates *et al.*, 2015), we fit a linear mixed effects model and fit a series of models, including a full and null model, to predict FGM concentrations as a function of four, two-way interactions between reproductive status (i.e. lactating, not-lactating) and the variables of total rainfall (July–August, 1-month and 2-months prior to capture), percent sand in surface soil, percent brush cover, body condition score and ordinal date, as well as each of these parameters as main effects. We included ordinal date (i.e. the number of days past October 1 of the capture year) in the analysis to account for the variation in lactating females as a function of capture date because deer captured later in the season are less likely to show evidence of lactation (Cherry *et al.*, 2016). Lastly, we included random effects with random intercepts for ranch and capture year. To avoid pseudoreplication from repeated measures, we omitted the second capture occasion from three individuals. We scaled and centred all model covariates and log transformed FGM concentrations to reduce positive skew in model residuals. We used the ‘dredge’ function from the ‘MuMIn’ package (Barton, 2023) to evaluate all possible combinations of variables used in the full model. Our hypothesis regarding carry-over effects was represented by models including rainfall during July and August. If these models received more support than models including static environmental variables or more recent rainfall (1-month and 2-months prior to capture), we would interpret that result as support for the hypothesis that previous exposure to environmental stressors would influence an individual’s current GC levels (i.e. physiological carry-over effect). To remove the negative bias AIC imposes for small sample sizes (Hurvich and Tsai, 1989), we used Corrected Akaike Information Criterion (AICc) to identify the most supported model. We evaluated the informative parameters within the most supported model using Satterthwaite’s method to estimate the degrees of freedom and compute *P*-values for all direct effects and interactions using the t-statistic (Kuznetsova *et al.* 2017).

## Results

**Figure 2:**
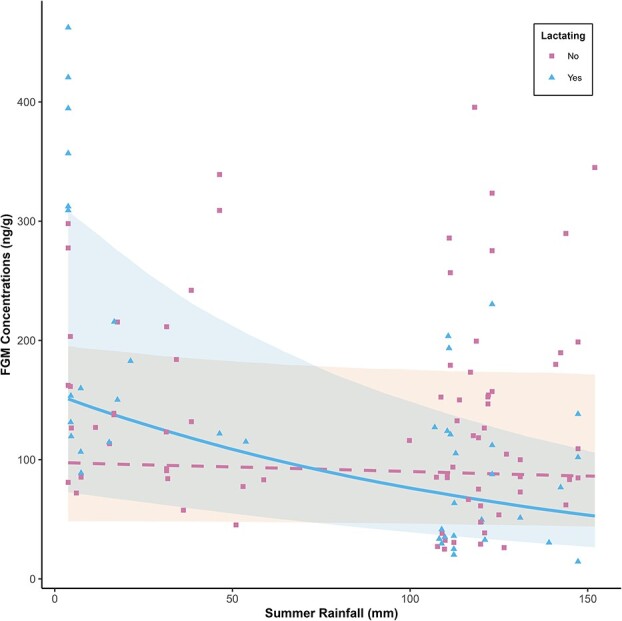
Model output depicting the relationship between FGM concentrations (ng/g) and summer (i.e. July and August) rainfall (mm) in lactating (solid line) and non-lactating (dashed line) female white-tailed deer on four East Foundation ranches during years 2015, 2016 and 2021. Bands around the predicted curves represent 95% confidence intervals. The triangles represent FGM concentrations of lactating female deer. The squares represent FGM concentrations of non-lactating female deer.

We found evidence reduced summer rainfall imparted long-term effects on female GC levels, but the effect of past environmental conditions depended on reproductive status. The most supported model predicting FGM concentrations included the interaction between summer rainfall and reproductive status (i.e. lactating, not lactating) ($\hat{\beta}$ = −0.32, SE +/− 0.08, *P* < 0.001, Tables 2 and 3). For every 1 cm decrease in summer rainfall, FGM concentrations increased 6.9% in lactating females in autumn; however, there was no effect of summer rainfall on FGM concentrations in non-lactating females in autumn (Fig. 2). We found some support for a model including percent brush cover ($\hat{\beta}$ = 0.17, SE ± 0.06, *P* < 0.001, Table 2). For every 1% increase in brush cover, FGM concentrations increased 1.44%. However, we found no support for models including other environmental conditions as models containing percent sand in soil, ordinal date, body condition score and current rainfall (i.e. 1- and 2-months prior to capture) were not competitive with the top model (Table 2).

**Table 2:** Candidate models predicting the relationship between FGM concentrations, rainfall and habitat characteristics on four East Foundation ranches

Model	K	AICc	$ \Delta $ AICc	w_i_
Lactation + Summer Rain + Lactation^*^Summer Rain	7	202.45	0	0.39
Brush	5	204.02	1.69	0.56
Null model	4	205.23	4.06	0.9
Rain 2-Months Prior to Capture	5	207.10	4.64	0.94
Rain 1-Month Prior to Capture + Brush	6	207.43	4.97	0.97

The models include our competitive models (i.e. AICc < 2), a null model and models for current rainfall (i.e. 1- and 2-months prior to capture). K is the number of variables. ΔAICc is the difference between the top performing model’s AICc and the lesser performing models. w_i_ is the cumulative model weight. Brush is percent brush cover. Summer Rain is total rainfall during July and August within years 2015, 2016 and 2021.

**Table 3:** Parameters estimates for the top performing linear regression model predicting log-FGM concentrations of reproductively mature female white-tailed deer as a function of reproductive status (i.e. lactating, not lactating), total rainfall in July and August, and the interaction between reproductive status and rainfall in July and August during autumn of 2015, 2016 and 2021 on the East Foundation’s Buena Vista Ranch, El Sauz Ranch, Santa Rosa Ranch and San Antonio Viejo Ranch in South Texas, USA. Our reference class was non-lactating females. Lactating = lactating female, summer rainfall = total rainfall during July and August within years of study, beta coefficient ($\hat{\beta}$), standard error (SE), degrees of freedom (DF), *t*-value and *P*-value

Parameters	$\hat{\beta}$	SE	DF	*t*-value	*P*-value
Intercept	4.51	0.34	2	13.43	0.004
Lactating	−0.08	0.09	114	−0.88	0.379
Summer Rainfall	−0.04	0.06	115	−0.72	0.471
Lactating^*^Summer Rainfall	−0.32	0.08	116	−3.61	<0.001

## Discussion

We found support for the hypothesis that previous exposure to environmental stressors would influence an individual’s current GC levels. Our findings show reduced summer rainfall was associated with an increase in FGM concentrations during autumn in lactating deer; however, summer rainfall had little effect on autumn FGM concentrations in non-lactating female deer. We found no support for the competing hypothesis that current conditions are more predictive of an individual’s GC levels. Our findings show autumn rainfall (i.e. 1-month and 2-months prior to capture) had no effect on FGM concentrations in reproductively mature female deer. Lastly, there was no effect of body condition on FGM concentrations, suggesting this physiological response was not mediated by nutritional stress. Our findings provide evidence past environmental stressors, specifically reduced seasonal rainfall, may impact current GC levels, particularly in those individuals most energetically vulnerable, such as reproductively active females.

The long-term impact of reduced summer rainfall on autumn FGM concentrations in lactating, but not non-lactating, female deer suggests an individual’s energy balance may play a pivotal role in the long-term consequences of environmental stressors on GC levels. Prolonged activation of the HPA axis after the stressor has ended may help in energy regulation to aid the individual in coping with and recovering from the costs associated with the stressor (Wingfield *et al.*, 1998). In our study, it is likely lactating female deer who experienced reduced summer rainfall had higher GCs in autumn because they may have still been in an energetic deficit (Gittleman and Thompson, 1988; Clutton-Brock *et al.*, 1989; Pekins *et al.*, 1998; Therrien *et al.*, 2008). The required energy input for lactation may not be met during times of reduced rainfall triggering the release of GCs to mobilize energy to finance reproduction with an individual’s own body stores (Mautz, 1978; Stephens *et al.*, 2014; Rödel *et al.*, 2016). This energy deficit may remain high despite deer being temporally separated from the summer conditions and having recovered from any nutritional deficits at the time of sampling in autumn, thus producing sustained elevations in GC levels to meet energetic demands. We also found FGM concentrations were not influenced by current rainfall conditions (i.e. 1- and 2-months prior to capture) despite considerable variation across years and populations. In South Texas, autumn rainfall is the primary driver of forage availability at the time of our sampling efforts (Box, 1960; Dodd and Holtz, 1972). Many studies have shown current nutritional resource availability can drive FGMs in free-living animals (Chapman *et al.*, 2007; Busch and Hayward, 2009; Ayres *et al.*, 2012; Wasser *et al.*, 2017; Hunninck *et al.*, 2020). In our study, the lack of effect of current conditions on FGMs, as compared to past conditions, may reflect the impact summer rainfall conditions impose on a lactating female deer’s energetic needs. While lactating females may still utilize currently available forage to finance the costs of reproduction, the amount of energy consumed from the landscape may be insufficient to meet the deficit reduced summer rainfall imposed on the individual’s energetic balance. Thus, GCs remain elevated. The lack of effect of more immediate rainfall on FGM concentrations in autumn emphasizes the importance of understanding and recognizing stressors that impact animals during vulnerable life history stages (i.e. early lactation).

We found marginal support for the model predicting FGM concentrations as a function of percent brush cover. FGMs rose with increasing percent brush cover. Brush in a semi-arid region provides white-tailed deer thermal refugia (Dykes, 2022), nutrition (Arnold and Drawe, 1979; Hewitt, 2011; DeYoung *et al.*, 2019) and predator avoidance (Kie and Bowyer, 1999). Although in South Texas, many carnivores select brush cover (Sergeyev *et al.*, 2023). Nonconsumptive effects of predators on prey physiology have been demonstrated across taxa (Sheriff *et al.*, 2010; Vitousek *et al.*, 2014; Hammerschlag *et al.*, 2017; Dulude-de Broin *et al.*, 2020). Therefore, it is possible deer who selected brush cover for concealment had higher GC levels because they experienced greater predation risk. Alternatively, brush may directly impact FGMs through digestion dynamics. In ruminants whose diets shifted to more woody vegetation, FGMs increased (Hunninck *et al.*, 2020), an effect likely attributed to energetic costs of digestion (Vangilder *et al.*, 1982). While we do not have dietary data for the individuals selecting brush cover, our results on the relationship between brush and FGMs are of interest and provide an area where additional research is needed.

FGM concentrations were not influenced by percent sand in the surface soils despite the reported relationship between this environmental attribute and deer productivity in our system (Rankins *et al.*, 2023b). This result was surprising given previous studies showed individuals living in high quality habitat had lower GC levels when compared to individuals in low quality habitat (Shipley *et al.*, 2022; Whipple *et al.*, 2022). For example, in impala (*Aepyceros melampus*) in the Serengeti, GC levels increased with declining vegetation greenness (measured by normalized difference vegetation index), which was attributed to reduced rainfall leading to a lack of nutrient-rich vegetation forcing impala to shift their diet to less nutrient-rich forage (Hunninck *et al.*, 2020). In American pika (*Ochotona princeps*), GC levels increased with reduced habitat quality, likely driven by the timing of snowmelt, spring green-up and nutritional availability (Whipple *et al.*, 2022). In our study, we saw stronger evidence for summer rain than stable environmental characteristics on FGM concentrations. These findings emphasize the importance of the quantity and timing of rainfall in a semi-arid environment such that the effect of habitat characteristics on FGMs was overshadowed by the predominant effect of rainfall (DeYoung *et al.*, 2019).

This is one of the first studies to examine how a past stressor may have long-term impacts on individual GC levels in a free-living, long-lived mammal. We found summer environmental conditions (i.e. reduced summer rainfall) significantly increased FGM concentrations in lactating, but not non-lactating deer in autumn, findings consistent with a physiological carry-over effect. Although our methodology and findings are robust, we acknowledge our approach depended on simplifying assumptions and that there may be confounding factors in our observational study. By selecting a 150-ha buffer around capture locations, we have simplified the dynamics of deer space use and movement. Nonetheless, we suggest our method is a reasonable proxy of space use based upon concurrent movement data from our system (Spencer *et al.*, 2024). Additionally, we determined reproductive status via evidence of lactation at the time of capture. This method is a coarse measure of reproduction that is insensitive to litter size and may have miscategorized female deer who were reproductively active during summer but were without a fawn at the time of capture due to early predation or abandonment (Cherry *et al.*, 2016). Despite this limitation, lactation status at the time of autumn captures is an appropriate measure of reproductive status because the highest percentage of fawn mortalities occur within the first three weeks of life, thus alleviating the energetic burden of lactation on the mother (Rohm *et al.*, 2007; Grovenburg *et al.*, 2011; Aubin *et al.*, 2022; Clevinger *et al.*, 2024). Lastly, there was high variability in the FGM concentrations of non-lactating individuals at all values of summer rainfall. Given we did not know the lactation status of individuals during summer, it is difficult for us to evaluate this variability in FGMs. Thus, future research should explore how maternal behaviour and movement decisions influence carry-over effects.

Understanding the long-term effects of extreme precipitation patterns on individual GC levels is critical given the predicted increase in drought frequency and severity with global climate change (Trenberth *et al.*, 2014; Spinoni *et al.*, 2018; Taylor *et al.*, 2021). Increased variability in rainfall patterns may impart more severe consequences than anticipated if individuals are still recovering from previous conditions, especially if the individual is in a more energetically vulnerable life history stage. These physiological carry-over effects may directly influence juvenile recruitment through impacts on reproductive investment (Smith *et al.*, 2004), ovulation (Cherry *et al.*, 2016), and the timing of breeding seasons (Goutte *et al.*, 2010, Zani and Stein, 2018), an important variable when considering harvest recommendations or recovery plans. In conclusion, understanding how previously experienced environmental stressors continue to impact an individual’s GC levels through physiological carry-over effects may provide insights into how animals cope with and respond to climate change and the associated increase in extreme environmental events.

## Supplementary Material

Hediger_SupplementaryMaterial_coae045

## Data Availability

The data sources that support the findings of this study are listed in the “Literature Cited” section. The data and code used to produce the figures and results in this manuscript can be found on GitHub: https://github.com/jhedigerdvm/Physiological-carry-over-effects
