# Supplementary material for: Physiological carry-over effects of variable precipitation are mediated by reproductive status in a long-lived ungulate
Source: Conserv Physiol. 2024 Jul 5;12(1):coae045. doi: 10.1093/conphys/coae045 (PMC11224986; doi:10.1093/conphys/coae045)
Supplement: Hediger_SupplementaryMaterial_coae045 [file hediger_supplementarymaterial_coae045.pdf]

SUPPLEMENTARY MATERIAL:

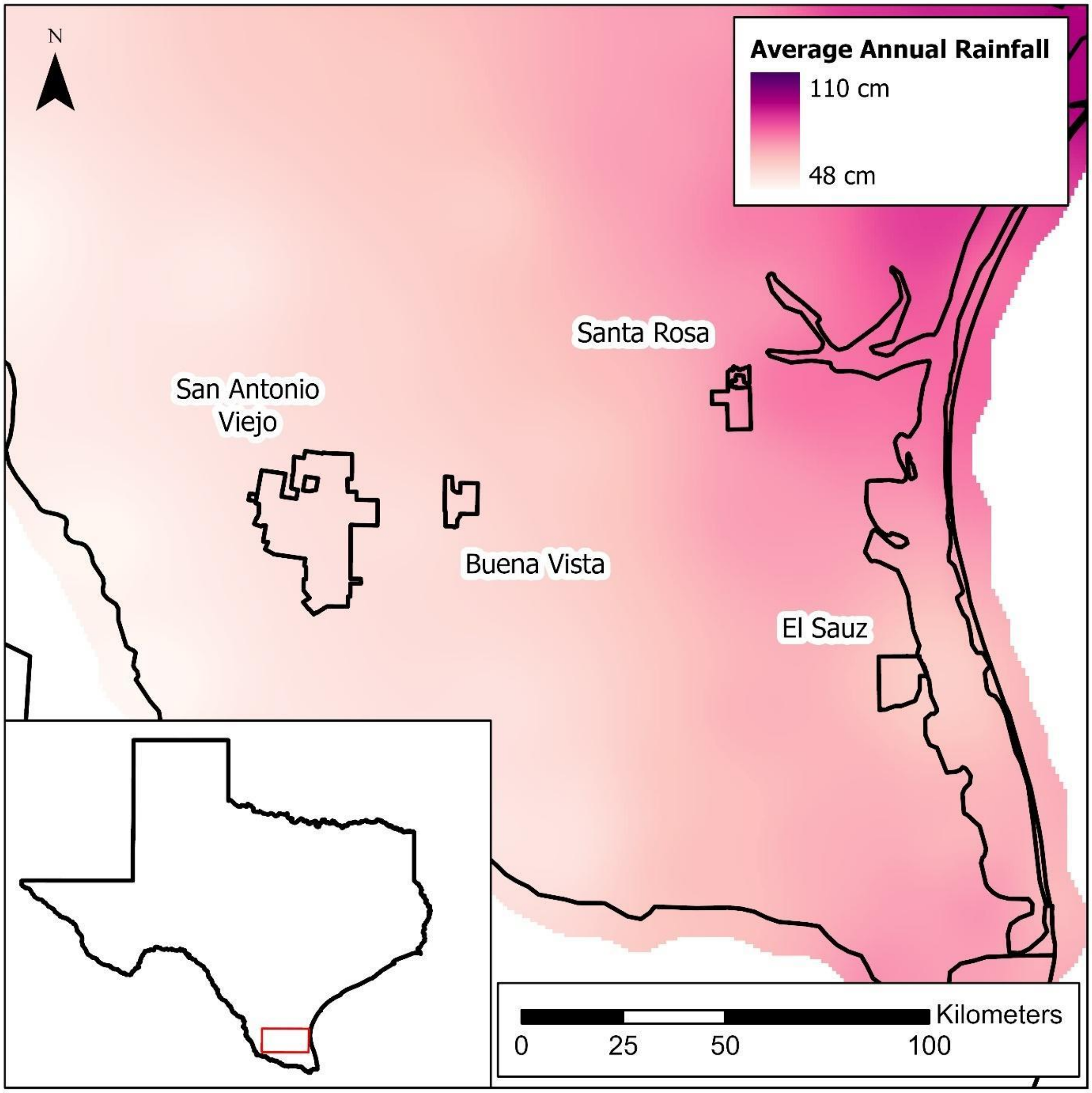

FIGURE 1 Map of South Texas, USA depicting the distribution of 30-year average annual rainfall (cm). Outlined in black are 4 East Foundation ranches: Buena Vista Ranch, El Sauz Ranch, Santa Rosa Ranch, and San Antonio Viejo Ranch.

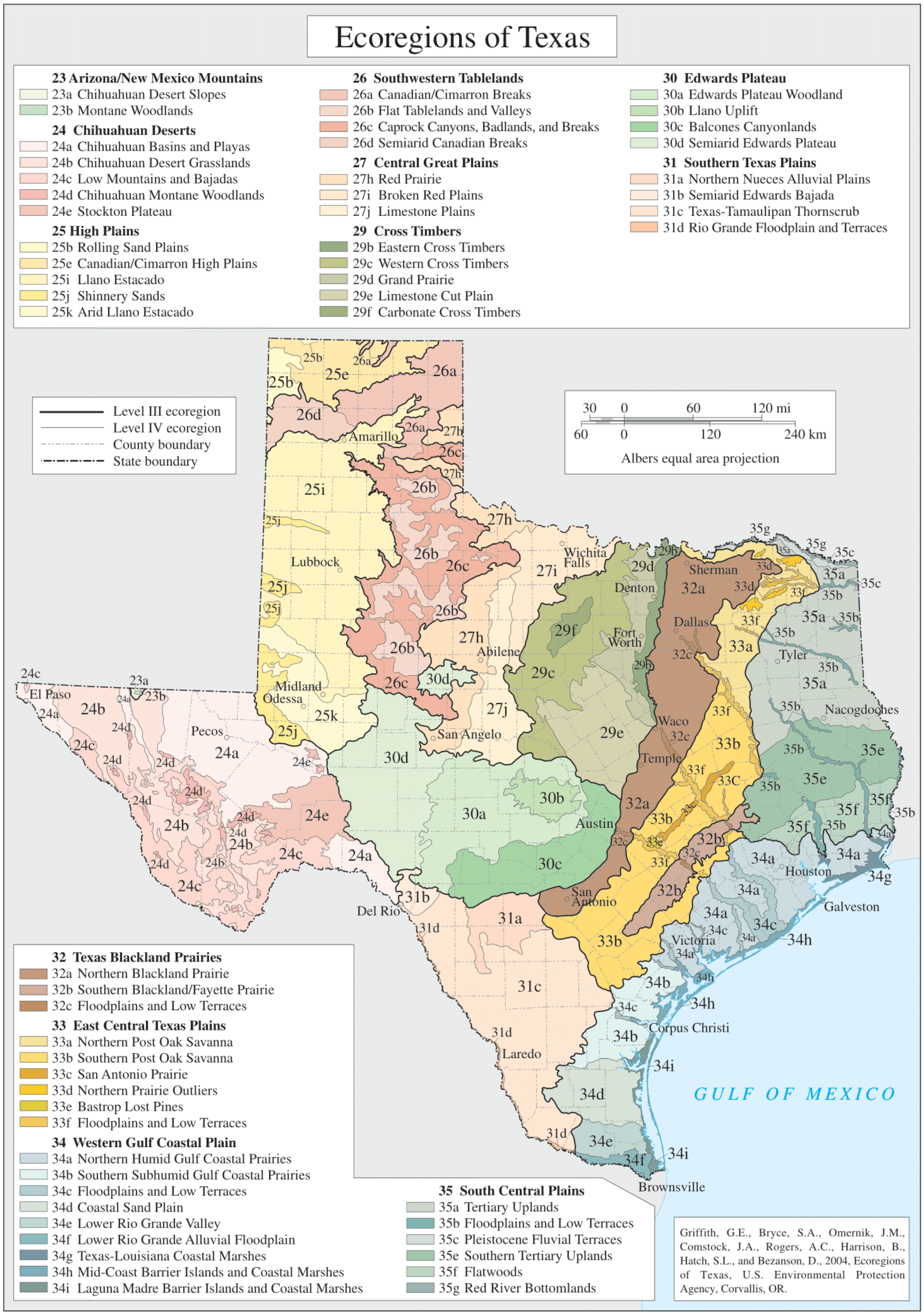
Ecoregions of Texas
23 Arizona/New Mexico Mountains
23a Chihuahuan Desert Slopes
23b Montane Woodlands
24 Chihuahuan Deserts
24a Chihuahuan Basins and Playas
24b Chihuahuan Desert Grasslands
24c Low Mountains and Bajadas
24d Chihuahuan Montane Woodlands
24e Stockton Plateau
25 High Plains
25b Rolling Sand Plains
25e Canadian/Cimarron High Plains
25i Llano Estacado
25j Shinnery Sands
25k Arid Llano Estacado
26 Southwestern Tablelands
26a Canadian/Cimarron Breaks
26b Flat Tablelands and Valleys
26c Caprock Canyons, Badlands, and Breaks
26d Semiarid Canadian Breaks
27 Central Great Plains
27h Red Prairie
27i Broken Red Plains
27j Limestone Plains
29 Cross Timbers
29b Eastern Cross Timbers
29c Western Cross Timbers
29d Grand Prairie
29e Limestone Cut Plain
29f Carbonate Cross Timbers
30 Edwards Plateau
30a Edwards Plateau Woodland
30b Llano Uplift
30c Balcones Canyonlands
30d Semiarid Edwards Plateau
31 Southern Texas Plains
31a Northern Nueces Alluvial Plains
31b Semiarid Edwards Bajada
31c Texas-Tamaulipan Thornscrub
31d Rio Grande Floodplain and Terraces
Level III ecoregion
Level IV ecoregion
County boundary
State boundary
30 0 60 120 mi
60 0 120 240 km
Albers equal area projection
Amarillo
Lubbock
Wichita Falls
Sherman
Denton
Dallas
Fort Worth
Tyler
Abilene
Midland
Odessa
El Paso
Pecos
San Angelo
Nacogdoches
Waco
Temple
Austin
San Antonio
Del Rio
Houston
Galveston
Victoria
Corpus Christi
Laredo
Brownsville
GULF OF MEXICO
32 Texas Blackland Prairies
32a Northern Blackland Prairie
32b Southern Blackland/Fayette Prairie
32c Floodplains and Low Terraces
33 East Central Texas Plains
33a Northern Post Oak Savanna
33b Southern Post Oak Savanna
33c San Antonio Prairie
33d Northern Prairie Outliers
33e Bastrop Lost Pines
33f Floodplains and Low Terraces
34 Western Gulf Coastal Plain
34a Northern Humid Gulf Coastal Prairies
34b Southern Subhumid Gulf Coastal Prairies
34c Floodplains and Low Terraces
34d Coastal Sand Plain
34e Lower Rio Grande Valley
34f Lower Rio Grande Alluvial Floodplain
34g Texas-Louisiana Coastal Marshes
34h Mid-Coast Barrier Islands and Coastal Marshes
34i Laguna Madre Barrier Islands and Coastal Marshes
35 South Central Plains
35a Tertiary Uplands
35b Floodplains and Low Terraces
35c Pleistocene Fluvial Terraces
35e Southern Tertiary Uplands
35f Flatwoods
35g Red River Bottomlands
Griffith, G.E., Bryce, S.A., Omernik, J.M., Comstock, J.A., Rogers, A.C., Harrison, B., Hatch, S.L., and Bezanson, D., 2004, Ecoregions of Texas, U.S. Environmental Protection Agency, Corvallis, OR.

FIGURE 2 Texas Ecoregion Map courtesy the United States Environmental Protection Agency. Our study took place on the South Texas Plains (31) and Coastal Sand Plain (34d).

TABLE 1 Summary rainfall (mm) estimates by East Foundation ranch and year from captured deer home ranges. Min = minimum, SD = standard deviation, Max = maximum, BV = Buena Vista Ranch, ES = El Sauz Ranch, SAV = San Antonio Viejo Ranch, SR = Santa Rosa Ranch

Ranch	Year	Min	Mean	SD	Max
BV	2015	5	6	1	7
BV	2016	119	120	1	122
ES	2015	100	111	6	127
ES	2016	29	37	6	46
SAV	2015	4	9	6	21
SAV	2016	111	132	13	147
SAV	2021	104	116	8	130
SR	2015	52	53	2	56

TABLE 2 Percentage of sand in the surface soils by East Foundation ranch and year for captured deer home ranges. Min = minimum, SD = standard deviation, Max = maximum, BV = Buena Vista Ranch, ES = El Sauz Ranch, SAV = San Antonio Viejo Ranch, SR = Santa Rosa Ranch

Ranch	Year	Min	Mean	SD	Max
SR	2015	74	75	1	76
BV	2015	62	65	2	69
BV	2016	64	66	1	66
ES	2015	54	60	5	70
ES	2016	55	62	5	70
SAV	2015	46	52	3	58
SAV	2016	50	55	3	60
SAV	2021	54	57	2	60

TABLE 3 Percentage of brush cover by East Foundation ranch and year for captured deer home ranges. Min = minimum, SD = standard deviation, Max = maximum, BV = Buena Vista Ranch, ES = El Sauz Ranch, SAV = San Antonio Viejo Ranch, SR = Santa Rosa Ranch

Ranch	Year	Min	Mean	SD	Max
SR	2015	13	19	5	27
BV	2015	15	30	10	44
BV	2016	16	25	7	32
ES	2015	12	22	7	33
ES	2016	18	27	5	35
SAV	2015	17	37	14	61
SAV	2016	14	26	9	45
SAV	2021	33	43	7	55
